# Supplementary material for: Splice-Junction-Based Mapping of Alternative Isoforms in the Human Proteome
Source: Cell Rep. Author manuscript; Available in PMC 2020 Jan 15. (PMC6961840; doi:10.1016/j.celrep.2019.11.026)
Supplement: 2 [file NIHMS1546469-supplement-2.zip › DF1/PXD006675/RightVentricle/RightVentricle_55_TPM1_AISEELDHALNDMTSGPMLK.pdf]

A

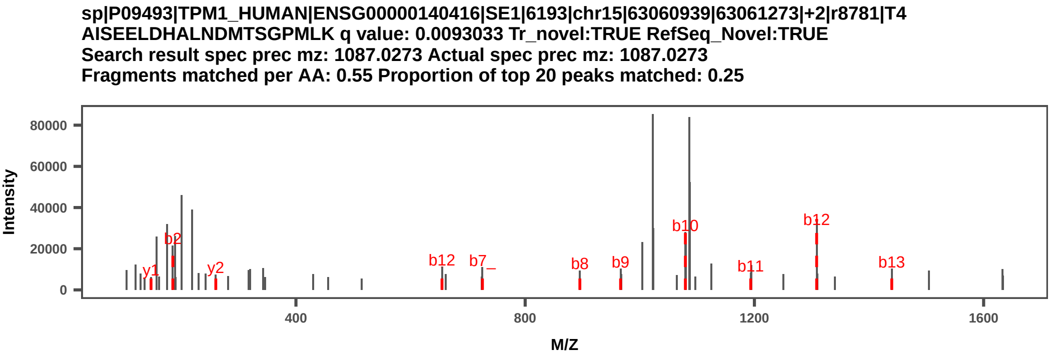
sp|P09493|TPM1_HUMAN|ENSG00000140416|SE1|6193|chr15|63060939|63061273|+2|r8781|T4
AISEELDHALNDMTSGPMLK q value: 0.0093033 Tr_novel:TRUE RefSeq_Novel:TRUE
Search result spec prec mz: 1087.0273 Actual spec prec mz: 1087.0273
Fragments matched per AA: 0.55 Proportion of top 20 peaks matched: 0.25
Intensity
80000
60000
40000
20000
0
y1
b2
y2
b12
b7_
b8
b9
b10
b11
b12
b13
400
800
1200
1600
M/Z

B

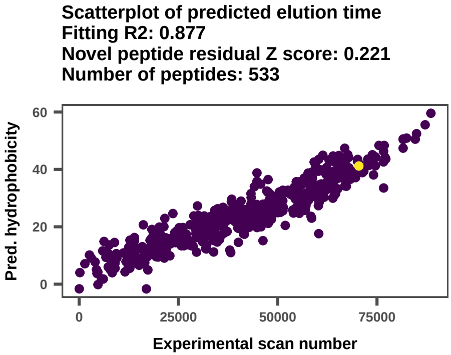
Scatterplot of predicted elution time
Fitting R2: 0.877
Novel peptide residual Z score: 0.221
Number of peptides: 533
Pred. hydrophobicity
60
40
20
0
0
25000
50000
75000
Experimental scan number

C

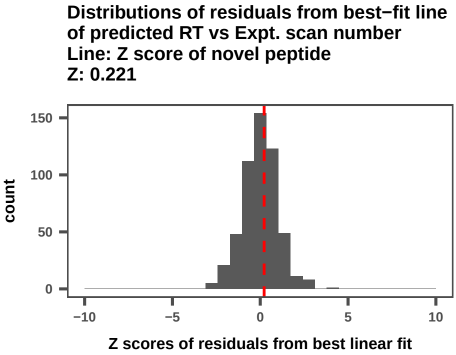
Distributions of residuals from best−fit line
of predicted RT vs Expt. scan number
Line: Z score of novel peptide
Z: 0.221
count
150
100
50
0
−10
−5
0
5
10
Z scores of residuals from best linear fit
